# Integrated Copy Number and Expression Analysis Identifies Profiles of Whole-Arm Chromosomal Alterations and Subgroups with Favorable Outcome in Ovarian Clear Cell Carcinomas

**DOI:** 10.1371/journal.pone.0128066

**Published:** 2015-06-04

**Authors:** Yuriko Uehara, Katsutoshi Oda, Yuji Ikeda, Takahiro Koso, Shingo Tsuji, Shogo Yamamoto, Kayo Asada, Kenbun Sone, Reiko Kurikawa, Chinami Makii, Otoe Hagiwara, Michihiro Tanikawa, Daichi Maeda, Kosei Hasegawa, Shunsuke Nakagawa, Osamu Wada-Hiraike, Kei Kawana, Masashi Fukayama, Keiichi Fujiwara, Tetsu Yano, Yutaka Osuga, Tomoyuki Fujii, Hiroyuki Aburatani

**Affiliations:** 1 Genome Science Division, Research Center for Advanced Science and Technology, The University of Tokyo, Tokyo, Japan; 2 Department of Obstetrics and Gynecology, The University of Tokyo, Tokyo, Japan; 3 Department of Pathology, The University of Tokyo, Tokyo, Japan; 4 Department of Obstetrics and Gynecology, Saitama Medical University International Medical Center, Saitama, Japan; 5 Department of Obstetrics and Gynecology, Teikyo University International, Tokyo, Japan; 6 Department of Obstetrics and Gynecology, National Center for Global Health and Medicine, Tokyo, Japan; Philipps University, GERMANY

## Abstract

Ovarian clear cell carcinoma (CCC) is generally associated with chemoresistance and poor clinical outcome, even with early diagnosis; whereas high-grade serous carcinomas (SCs) and endometrioid carcinomas (ECs) are commonly chemosensitive at advanced stages. Although an integrated genomic analysis of SC has been performed, conclusive views on copy number and expression profiles for CCC are still limited. In this study, we performed single nucleotide polymorphism analysis with 57 epithelial ovarian cancers (31 CCCs, 14 SCs, and 12 ECs) and microarray expression analysis with 55 cancers (25 CCCs, 16 SCs, and 14 ECs). We then evaluated *PIK3CA* mutations and *ARID1A* expression in CCCs. SNP array analysis classified 13% of CCCs into a cluster with high frequency and focal range of copy number alterations (CNAs), significantly lower than for SCs (93%, *P* < 0.01) and ECs (50%, *P* = 0.017). The ratio of whole-arm to all CNAs was higher in CCCs (46.9%) than SCs (21.7%; *P* < 0.0001). SCs with loss of heterozygosity (LOH) of *BRCA1* (85%) also had LOH of *NF1* and *TP53*, and LOH of *BRCA2* (62%) coexisted with LOH of *RB1* and *TP53*. Microarray analysis classified CCCs into three clusters. One cluster (CCC-2, *n* = 10) showed more favorable prognosis than the CCC-1 and CCC-3 clusters (*P* = 0.041). Coexistent alterations of *PIK3CA* and *ARID1A* were more common in CCC-1 and CCC-3 (7/11, 64%) than in CCC-2 (0/10, 0%; *P* < 0.01). Being in cluster CCC-2 was an independent favorable prognostic factor in CCC. In conclusion, CCC was characterized by a high ratio of whole-arm CNAs; whereas CNAs in SC were mainly focal, but preferentially caused LOH of well-known tumor suppressor genes. As such, expression profiles might be useful for sub-classification of CCC, and might provide useful information on prognosis.

## Introduction

Epithelial ovarian cancer is a leading cause of death resulting from gynecological malignancies, and is characterized by high recurrence and poor survival rates [1]. Ovarian cancer is a heterogeneous group of diseases, and can be classified into four major histological subtypes: serous carcinoma (SC), mucinous carcinoma (MC), endometrioid carcinoma (EC), and clear cell carcinoma (CCC). Genome-wide copy number alterations (CNAs) and gene expression profiles for ovarian tumors have been constructed from analyses consisting mainly of SC tumor samples, as high-grade SC is the most common histological subtype and accounts for approximately 60–70% of all the ovarian cancers [2]. The Cancer Genome Atlas Research Network (TCGA) recently provided a more integrated genomic analysis of HG-SC [3]. High-grade SC is characterized by highly prevalent *TP53* mutations, statistically recurrent mutations of other tumor suppressor genes (*NF1*, *RB1*, and *BRCA1/2*), and CNAs at specific loci. *TP53* inactivation, followed by *BRCA* inactivation, induces chromosomal instability and focal CNAs [4]. *BRCA1/2* mutations (germline or somatic) and *BRCA1* promoter hypermethylation have prevalences of 21% and 11%, respectively [3]. Copy number loss of *BRCA2* and *RB1*, as well as *TP53* and *BRCA1* CNAs, were potentially correlated with each other in BRCA-mutated ovarian cancers [5]. However, the contribution of BRCA copy number loss and its correlation with CNAs in other tumor-suppressor genes in various types of (mainly sporadic) ovarian cancer remain unclear.

CCC is the second leading cause of death from ovarian cancer, with an increased incidence in Asia [2,6]. Notably, the prevalence of CCC in ovarian cancer is approximately 25% in Japan. CCC tumorigenesis is suggested to proceed in a stepwise fashion−starting with endometriosis, then progressing through atypical endometriosis or adenofibroma, and culminating in a carcinoma [7–9]. Owing to the low response rate observed with conventional platinum-taxane chemotherapy, the clinical outcome of CCC is generally poor, even when diagnosed at an early stage [10–12]. As such, it is crucial to identify key subgroups that may be more sensitive to other types of therapeutics. Previous microarray analyses have identified a CCC expression profile that is distinct from other epithelial ovarian cancer histotypes [13–15]. HNF-1beta and oxidative stress-related genes are upregulated in CCC [16–18]. The numbers of CNAs in CCC are similar to those in low-grade SC and much less than those found in high-grade SC [19]. Notably, a gain on chromosome 20q13.2, which harbors a potential oncogene, *ZNF217*, has been suggested to be a poor prognostic factor in CCC [20]. Recently, a sub-classification of CCC by CNAs has suggested that those with extensive chromosomal instability might be associated with poor prognosis [21]. Mutations of *ARID1A* and *PIK3CA* are more frequent (>40%) in CCC than in other histological types [3,22–26]. The histotype-specificity of CNAs was previously reported in ovarian cancer by Huang et al. [27]. These authors mainly focused on histotype-specific candidate driver genes with CNAs, including *ERBB2* in mucinous histotypes and *TPM3* in endometrioid histotypes [27]. However, a classification of CCC based on the combined analysis of gene expression and chromosomal instability has not been performed and additional integrated genomic profiling is required to elucidate the tumor biology and identify biomarkers for predicting CCC.

In this study, we focused on the distinguishing features of CCC, compared to SC and EC. Using data obtained from single-nucleotide polymorphism (SNP) arrays and gene expression arrays, we have characterized the genomic profiles of CCC to further explore prognostic signatures in CCC.

## Materials and Methods

### Tumor samples and genomic DNA

Surgical samples were obtained from 57 patients (31 CCCs, 14 SCs, and 12 ECs) for copy number analysis and from 55 patients for expression arrays (25 CCCs, 16 SCs, and 14 ECs), using samples from patients who underwent tumor resections at the University of Tokyo Hospital. In total, 80 patients were recruited for this study, and 32 patients (20 CCCs, 7 SCs, and 5 ECs) overlapped between the copy number analysis and expression array studies (S1 Table). Histologies were determined by one pathologist and independently confirmed by a second pathologist. Only SCs classified as high-grade were included in this study. All patients received primary surgery, including hysterectomy, bilateral salpingo-oophorectomy, and omentectomy, together with systematic lymphadenectomy (when mass reduction was completely or optimally achieved). The patients with stage Ic–IV received six to eight cycles of adjuvant chemotherapy (paclitaxel and carboplatin). Chemosensitivity was evaluated in patients with residual or recurrent measurable disease. All patients provided written informed consent for the research use of their samples, and the collection and use of tissues for this study were approved by the Human Genome, Gene Analysis Research Ethics Committee at the University of Tokyo.

The fresh-frozen tumors were embedded in OCT (optimum cutting temperature) compound, and the 4-mm thick tissue sections were stained with hematoxylin and eosin. Tissue sections with a high proportion of carcinoma cells (>50%) were reviewed by a pathologist and selected for DNA and total RNA extraction. Genomic DNA was isolated from the tumor sections or lymphocyte pellets using a QIAamp DNA Mini Kit (Qiagen, Valencia, CA, USA), according to the manufacturer’s specifications. As a control for copy number analysis, paired genomic DNA was also extracted from blood samples of 57 patients.

### SNP array

SNP arrays were performed for 57 clinical samples with paired tumor DNA and normal DNA using a GeneChip Human Mapping 250K Nsp Arrays (Affymetrix, Santa Clara, CA, USA). Experimental procedures for GeneChip arrays were performed according to the GeneChip Expression Analysis Technical Manual (Affymetrix).

### Genome imbalance map

The genome imbalance map (GIM) algorithm was applied to raw data of endometrial cancer and peripheral blood obtained from SNP arrays. We previously reported that tissue sections comprised of ≥50% epithelial-derived tumor are sufficient for the GIM algorithm [28]. The basic concept of GIM involves normalization of probe level signals, as described previously [28,29]. Briefly, the signal intensity ratio between the raw signal intensity from the cancer and paired normal samples was calculated from the perfect match probes for each SNP locus, using the median values considering the median after omitting the highest and lowest values. For allele-specific copy number analysis in this GIM algorithm, the relative ratios of 0.5, 1, and 1.5, theoretically correspond to 0, 1, and 2 copies, respectively. We detected allele-specific CNAs, using the cut-off relative ratio of >1.3 (1.6 copies) for gain and <0.7 (0.4 copies) for loss in each region. A region with a total copy number of three or more without loss of heterozygosity (LOH) is considered as a copy numbers gain, a region with loss of both alleles as homozygous deletion, and a region including hemizygous deletion with a gain of the opposite allele as copy number neutral LOH (CNN LOH). The type of CNAs were classified into focal (length < 98% of a chromosome arm) and whole-arm CNAs (length >98% of a chromosome arm.)

### RNA extraction and microarray analysis

Tissues were lysed directly in TRIzol reagent (Invitrogen, Carlsbad, CA) and homogenized. Total RNA was extracted according to the manufacturer’s instructions. Fifty-five ovarian cancer tissues were analyzed on HG-U133 Plus 2.0 arrays (Affymetrix) containing 54,675 probes for human genes. Microarray analysis was performed as described previously [30]. For global normalization, the average signal in an array was given a value of 100. Gene expression data were deposited within the NCBI Gene Expression Omnibus (GEO), Accession No: GSE65986.

### Clustering study

An unsupervised hierarchical clustering algorithm was used to classify clusters on the basis of the Euclidean distance for dissimilarities between the SNP array data of the samples. The calculations were performed in Cluster3.0, Java TreeView, and the algorithm parameters were set to Measurement = Euclidean, Linkage = Complete.

The same algorithm was also used to identify clusters based on the Euclidean distance for dissimilarities in gene expression between the tumor and normal samples. The calculations were performed using GeneSpring GX 7.3 (Agilent, Santa Clara, CA). From the 54,675 probes in the HG-U133 Plus 2.0 array, we selected 2640 probes that produced a maximum signal of ovarian cancer samples ≥100, an average signal ≥10, and a coefficient of variation ≥0.3.

### k-means clustering and class prediction

k-means clustering was performed as follows: (i) changing the sample order 1,000 times by selecting randomly 3,000 genes, (ii) identifying samples that were classified in the same cluster together, and (iii) repeating steps (i) and (ii) for 2 to 10 k groups. Non-negative matrix factorization was optimized on basis of a consensus matrix by k-means clustering for 2 to 10 k groups, and the lowest approximation error across multiple runs was calculated [31].

### Reverse transcription and quantitative real-time PCR (real-time RT PCR)

Reverse transcription and quantitative real-time PCR (real-time RT PCR) cDNAs were synthesized from total RNAs in 11 CCC samples by using the Super Script III First-Strand Synthesis SuperMix (Invitrogen, Carlsbad, CA) [32]. The mRNA levels of *UGT1A* (*UGT1A6* and *UGT1A10*) were measured by quantitative real-time RT-PCR, using the One Step SYBR PrimeScript RT-PCR Kit (TaKaRa Bio. Inc., Tokyo, Japan) in a Light Cycler instrument (Roche Applied Science, Mannheim, Germany), as described previously [33]. The sequences of the primer pairs used are as follows: *UGT1A6*: 5'-TGG GAT CAA TGG TCT CAG AAA TTC-3' (forward) and 5'-CGT GTT GTT CGC AAG ATT CGA TG-3' (reverse); and *UGT1A10*: 5'-GAA AGC ACA GGC ACA AAG TAT A-3' (forward) and 5'-GGG AGG GAG AAA TAT TTA GCA AC-3' (reverse).

### Mutational analysis of *PIK3CA* and immunohistochemistry of *ARID1A*

Mutations in *PIK3CA* (exon 9 and 20) were analyzed as described previously [34,35]. Immunohistochemical analysis of 21 CCCs was carried out on 4-μm whole tissue sections. The peptide sequence for the anti-ARID1A antibody (HPA005456; Sigma-Aldrich, St. Louis, MO) has been described previously [36]. Antigen retrieval was performed by placing sections in a citrate buffer (pH 6.0) and autoclaving at 120°C for 10 minutes. Sections were then incubated with the anti-rabbit IgG antibody overnight at 4°C. A positive reaction was detected using the EnVision+System (Dako, Carpinteria, CA). Tumor stromal cells served as positive internal controls and only nuclear staining was scored. A previous study showed that loss of nuclear expression correlates with mutation of the gene [25]. Hence, absence of nuclear staining (diffuse or focal) was considered positive for gene mutation.

### Statistical analysis

The association of variables related to clinical characteristics was evaluated by Fisher’s exact test. The *P* values obtained in all tests were considered significant at *P* < 0.05. Survival curves were constructed using the Kaplan–Meier method and compared with a log-rank test. The analyses were carried out using the JMP 9 statistics package (SAS Institute, Cary, NC). Multivariate analysis was conducted using Cox’s proportional hazard model.

## Results

### SNP array genotyping distinguishes histology-related subgroups according to status of chromosomal instability

**Fig 1:**
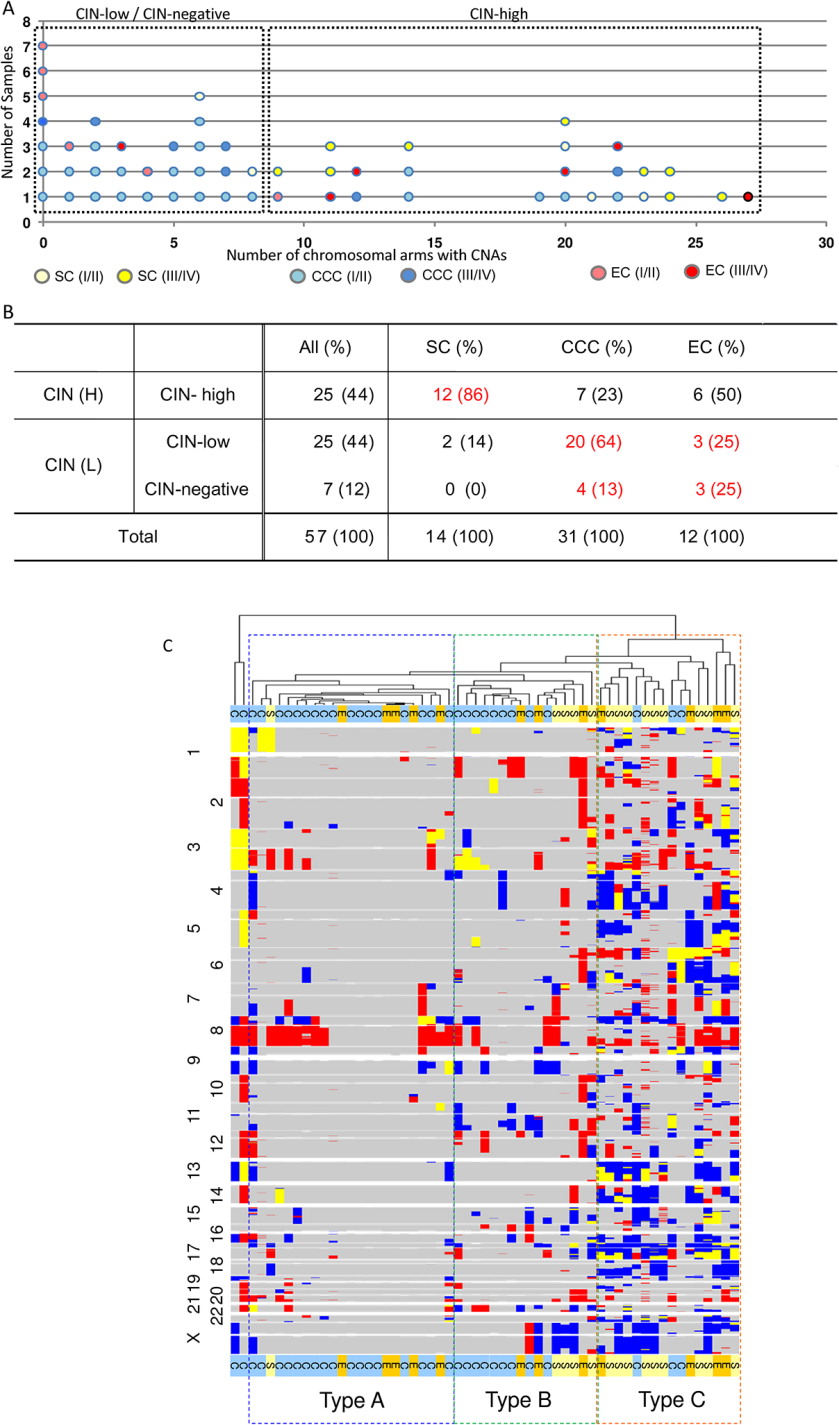
SNP profiling discriminates histology-related subgroups based on chromosomal instability status. Chromosomal instability status (CIN) according to the number of allele-specific copy number alterations (CNAs) and copy number neutral loss of heterozygosity (CNN LOH), using a human mapping 250K single nucleotide polymorphism (SNP) array with paired tumor DNA and normal DNA. CNAs were divided into three subgroups: CIN-high (≥9 arms with CNAs), CIN-low (1–8 arms with CNAs), and CIN-negative (0 CNAs). (A) Details of number of chromosomal arms with CNAs in each tumor of three histological subtypes (serous carcinomas, SC; clear cell carcinomas, CCC; endometrioid carcinomas, EC). Stage I/II and stage III/IV are colored differently. (B) Correlation between CIN status and histological subtypes. (C) Overview of CNAs by running SNP arrays with 57 ovarian cancer samples. Hierarchical clustering based on the Euclidean distance for dissimilarities is shown. The type A cluster includes tumors with a broad range and low frequency of CNAs, whereas the type B cluster includes tumors with a focal range and high frequency of CNAs. C, E, and S indicate clear cell carcinoma, endometrioid carcinoma, and serous carcinoma, respectively.

First, we evaluated chromosomal instability (CIN) in 57 ovarian carcinoma samples with paired DNA (tumor and normal) by SNP arrays (31 CCCs, 12 ECs, and 14 high-grade SCs). The median follow-up time is 36 months (4–144 months). All 57 samples were analyzed for allele-specific copy numbers and total copy numbers. Representative SNP array “karyograms” of each tumor are shown in S1 Fig. The Y-axis values for LOH in the karyograms of SNP array were comparable among the samples tested, suggesting that the tumor ratio in this study was suitable for analysis (S1 Fig). Fig 1A shows the number of chromosomal arms with CNAs for each sample. An overview of genomic imbalances sorted by histology is shown in S2 Fig. As shown in Fig 1A, we defined the CIN status based on the distribution of chromosomal arms with CNAs and divided the samples into three subgroups: CIN-high (≥9 arms with CNAs), CIN-low (1–8 arms with CNAs), and CIN-negative (0 CNAs). We also focused on relationships between CIN status and histological subtypes (CCC, EC, and SC). The ratio of CIN-high was significantly higher (*P* < 0.001 by Fisher’s exact test) in SCs (86%) than in CCCs (23%) (Fig 1B). The ratio of CIN-high in ECs was 50% (6/12). In ECs, 5 of the 6 advanced stage (stage III/IV) tumors were CIN-high (83%), whereas 1 of the 6 early stage (stage I/II) tumors was CIN-high (17%) (*P* = 0.080).

As the range and frequency of CNAs are distinct in each tumor, we structured a hierarchical clustering based on the Euclidean distance for dissimilarities in the SNP array data (Fig 1C). Type A (*n* = 21) was a cluster with broad range and low frequency of CNAs, type B (n = 16) was a cluster with broad range and low to high frequency of CNAs, and type C (*n* = 16) was a cluster with focal range and high frequency of CNAs. Twenty-six of 31 CCCs (84%) were classified into type A/B, but 5 out of 14 SCs (36%) were in type A/B (*P* = 0.0038). The type C cluster included 9 of 14 SCs (64%), 3 of 31 CCCs (10%), and 4 of 12 ECs (33%) (Fig 1C). The ratio of type C tumors was significantly lower in CCCs than in SCs (*P* < 0.001).

### Frequent whole-arm CNAs in CCCs

As the type A cluster includes whole-arm CNAs in various chromosomes, we compared the ratio of whole-arm CNAs between CCC and SC. We evaluated the ratio of whole-arm CNAs among all the CNAs of each chromosome in these two histological types (Table 1). The average number of CNAs in 31 CCCs was 6.8, compared with an average of 17.8 CNAs in 13 SCs. The overall ratio of whole-arm CNAs among all the CNAs in CCCs was 46.9% (99/211), which was significantly higher than in SCs (21.6%, 50/231) (*P* < 0.0001). Whole-arm CNAs in SCs were more frequent in shorter chromosomes, such as 17p, 17q, 18p, 18q, Xp, and Xq. In contrast, whole-arm CNAs in CCCs were frequent in longer chromosomes (from chromosomes 1 to 16) (Table 1). We analyzed the ratio of whole-arm CNAs for CCCs and SCs in the publicly available data set GSE30311 (deposited in GEO) and confirmed that the overall ratio of whole-arm CNAs among all CNAs was significantly higher in CCCs (64.4%: 125/194) than in SCs (20.4%: 227/1110) (*P* <0.0001). Thus, the chromosomes with frequent whole-arm CNAs were distinct between SC and CCC.

**Table 1:** Ratio of whole arm CNAs among all the CNAs in serous and clear cell carcinomas.

	SC (n = 14)		CCC (n = 31)	
Chr.	arm change	non-arm change	arm change	non-arm change
1p	1(20%)	4(80%)	3(50%)	3(50%)
1q	1(33%)	2(67%)	2(33%)	4(67%)
2p	0(0%)	4(100%)	2(50%)	2(50%)
2q	0(0%)	4(100%)	1(33%)	2(67%)
3p	0(0%)	5(100%)	3(50%)	3(50%)
3q	2(25%)	6(75%)	5(46%)	6(54%)
4p	1(25%)	3(75%)	2(40%)	3(60%)
4q	1(10%)	8(89%)	2(67%)	1(33%)
5p	0(0%)	2(100%)	2(67%)	1(33%)
5q	1(17%)	4(80%)	1(50%)	1(50%)
6p	1(20%)	4(80%)	3(100%)	0(0%)
6q	1(17%)	5(83%)	1(20%)	4(80%)
7p	1(13%)	7(100%)	1(25%)	3(75%)
7q	0(0%)	4(100%)	1(20%)	4(80%)
8p	1(10%)	8(89%)	7(50%)	7(50%)
8q	1(11%)	8(89%)	12(75%)	4(25%)
9p	0(0%)	4(100%)	2(33%)	4(67%)
9q	0(0%)	3(100%)	6(67%)	3(33%)
10p	0(0%)	3(100%)	1(100%)	0(0%)
10q	0(0%)	3(100%)	1(100%)	0(0%)
11p	0(0%)	4(100%)	2(67%)	1(33%)
11q	0(0%)	5(100%)	2(25%)	6(75%)
12p	0(0%)	2(100%)	4(67%)	2(33%)
12q	0(0%)	5(100%)	1(33%)	2(67%)
13q	2(25%)	6(75%)	4(80%)	1(20%)
14q	2(22%)	7(78%)	2(67%)	1(33%)
15q	0(0%)	7(100%)	1(14%)	6(86%)
16p	1(20%)	3(75%)	2(67%)	1(33%)
16q	1(17%)	4(80%)	5(50%)	5(50%)
17p	7(58%)	5(42%)	0(0%)	6(100%)
17q	4(33%)	8(67%)	1(20%)	4(80%)
18p	4(80%)	1(20%)	0(0%)	0(0%)
18q	4(43%)	3(57%)	0(0%)	1(100%)
19p	0(0%)	5(100%)	0(0%)	4(100%)
19q	0(0%)	3(100%)	1(25%)	3(75%)
20p	1(33%)	2(67%)	1(25%)	3(75%)
20q	1(14%)	6(86%)	3(38%)	5(62%)
21q	1(17%)	4(80%)	6(86%)	1(14%)
22q	1(14%)	6(87%)	0(0%)	4(100%)
Xp	5(63%)	2(29%)	3(100%)	0(0%)
Xq	5(63%)	2(29%)	3(75%)	1(25%)

### Chromosomal CNAs and histological subtypes in ovarian carcinomas

Copy number gains on chromosome arms and the chromosomal regions with these gains are listed in S2 and S3 Tables, respectively. Gains on 8q22.1–24.13 and 8q24.21–24.3 overlapped among all the three histological types. These loci cover several known oncogenes, including *CCNE2* (cyclin E2) and *MYC*. The ‘breakpoint’ region of gains on chromosome 8 is distinct between CCC and the other histological types. Chromosome 8q11.23 is the most common breakpoint in SC and EC, whereas 8p11.21 is most common in CCC. Thus, gains of 8p11.21–q11.23 are predominantly observed in CCCs (S3 Table). The most frequent regions of copy number gains (>50%) in SCs were located on chromosomes 3q (62%), 8q (69%), and 10q (54%). Gains of these chromosomes were also detected in CCCs and ECs, but at lower frequency. Gains on 20q13.13–13.33, including *ZNF217*, *PTPN1*, and *AURKA*, were also commonly observed in all the histological types. Gains of *PIK3CA*, *AKT1*, *AKT3*, *IGF1R*, and *FGF12* in the receptor tyrosine kinase (RTK)/Ras/PI3K pathway were more commonly detected in SCs at ≥36%.

Losses in SCs were frequent on chromosomes 4q (69%), 6q (46%), 8p (62%), 13q (62%), 14q (54%), 15q (46%), 17p (92%), 17q (85%), 18q (54%), 22q (46%), Xp (54%), and Xq (54%) (S2 Table). CNN LOH and homozygous deletions were less common in CCCs than in SCs (S2 Table). In particular, LOH of well-known tumor suppressor genes was especially prevalent in SCs, not in CCCs. The ratio of LOH, including CNN LOH, in SCs was 92% for 17p13.3 (*TP53*), 62% for 13q12.11–q31.1 (*RB1* and *BRCA2*), and 85% for 17q24.1–25.3 (*NF1* and *BRCA1*) (Table 2). All the SCs with LOH of *BRCA1* showed coexistent LOH of *NF1* and *TP53*, whereas all those with LOH of *BRCA2* harbored coexistent LOH of *RB1*, *NF1*, *BRCA1*, and *TP53* (Table 2). Thus, LOH of *BRCA* genes generally coexists with LOH of other tumor suppressor genes, such as *TP53*, *RB1*, and/or *NF1* in SCs. LOH at 4q28.1–35.2 was also frequent in SCs (50%); this region includes the loci of tumor suppressor *FBXW7* (F-box and WD repeat domain containing 7) and *FAT1* (FAT tumor suppressor homolog 1).

**Table 2:** LOH of major tumor suppressor genes in each histology.

	Gene	*TP53*	*NF1*	*BRCA1*	*RB1*	*BRCA2*
	Chr	17	17	17	13	13
	Cytoband	17p13.1	17q11.2	17q21.2	13q14.2	13q13.2
**n = 13**	SC-1	LOH	LOH	LOH	LOH	LOH
	SC-2	LOH	LOH	LOH	LOH	LOH
	SC-3	LOH	LOH	LOH	LOH	LOH
	SC-4	LOH	LOH	LOH	LOH	LOH
	SC-5	LOH	LOH	LOH	LOH	LOH
	SC-6	LOH	LOH	LOH	LOH	LOH
	SC-7	CNN LOH	LOH	LOH	LOH	LOH
	SC-8	CNN LOH	CNN LOH	CNN LOH	LOH	LOH
	SC-9	LOH	LOH	LOH	nl	nl
	SC-10	LOH	CNN LOH	LOH	nl	nl
	SC-11	CNN LOH	LOH	LOH	nl	nl
	SC-12	CNN LOH	nl	nl	nl	nl
	SC-13	nl	nl	nl	nl	nl
**n = 31**	CCC-1	LOH	nl	nl	LOH	LOH
	CCC-2	LOH	nl	nl	LOH	LOH
	CCC-3	LOH	nl	nl	LOH	nl
	CCC-4	LOH	nl	nl	nl	nl
	CCC-5	LOH	nl	nl	nl	nl
	CCC-6	LOH	nl	nl	nl	nl
	CCC-7	nl	nl	nl	LOH	LOH
	CCC-8	nl	nl	nl	CNN LOH	CNN LOH
	Other 23 cases (CCC 9–31)	nl	nl	nl	nl	nl
**n = 13**	EC-1	LOH	LOH	LOH	CNN LOH	LOH
	EC-2	LOH	nl	nl	nl	nl
	EC-3	LOH	nl	LOH	nl	nl
	EC-4	CNN LOH	LOH	CNN LOH	LOH	nl
	EC-5	CNN LOH	LOH	LOH	nl	nl
	Other 8 cases (EC6-13)	nl	nl	nl	nl	nl

nl: no copy number alterations, CNN LOH: Copy number neutral loss of heterozygosity.

### Hierarchical clustering by expression array in ovarian carcinomas

**Fig 2:**
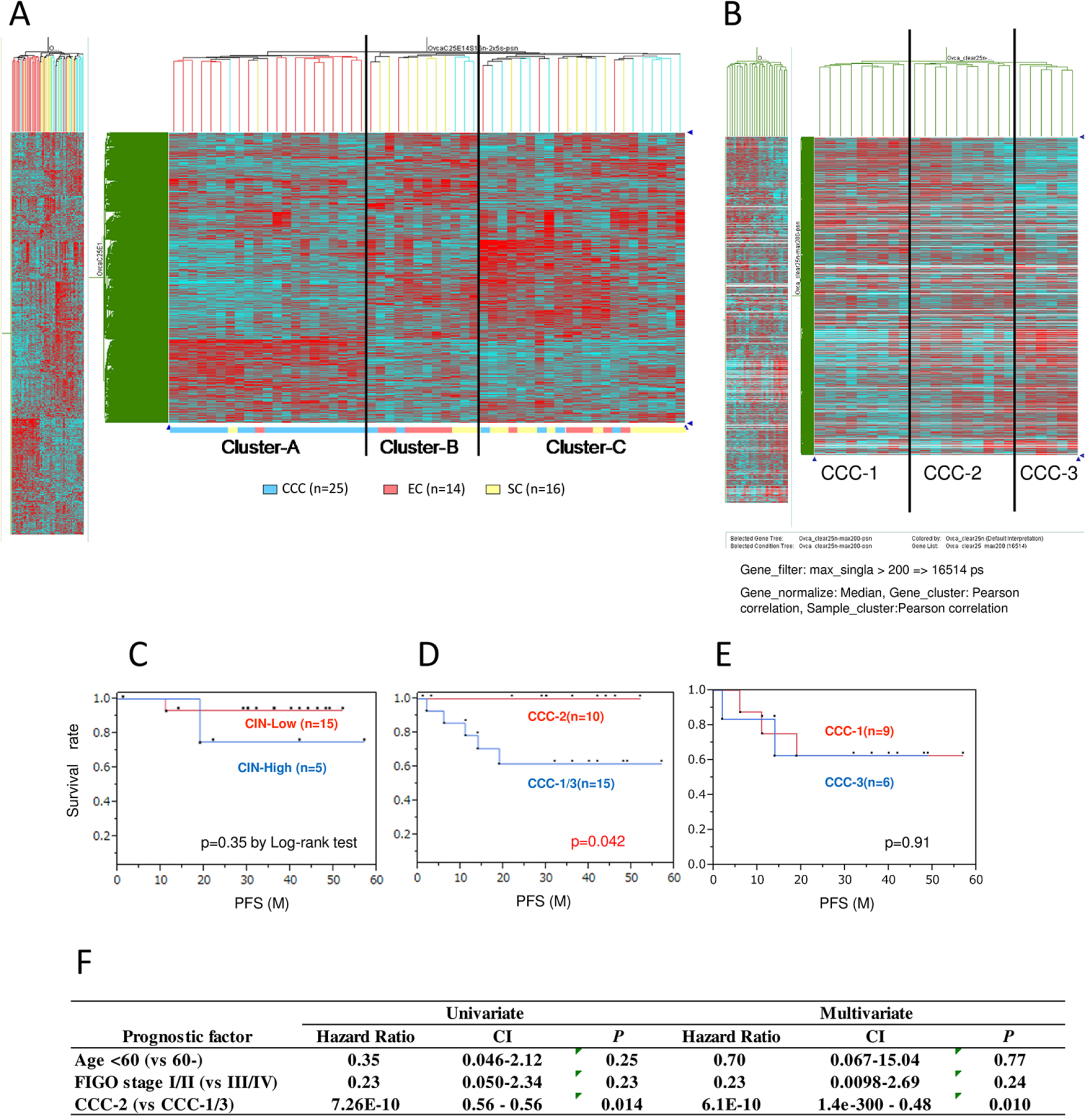
Unsupervised hierarchical clustering by gene expression array analysis in ovarian carcinomas. Microarray gene expression profiling was performed in 55 ovarian carcinomas (16 serous carcinomas [SCs], 25 clear cell carcinomas [CCCs], 14 endometrioid carcinomas [ECs]), followed by sub-clustering of the 25 CCCs. (A) Clustering of the 55 ovarian carcinomas using HG-U133 Plus 2.0 arrays. Clusters A, B, and C predominantly include CCC, EC, and SC, respectively. (B) Twenty-five CCCs were subdivided into three groups (CCC-1, CCC-2, and CCC-3) according to the hierarchical clustering. Nine (36%) tumors were classified as CCC-1, ten (40%) as CCC-2, and six (24%) as CCC-3. (C–E) Kaplan–Meier analysis according to the CIN status (C) and sub-classification of CCC (D and E). Cluster CCC-2 had a significantly favorable prognosis, compared with clusters CCC-1 and CCC-3 (D). (F) Multivariate analysis in 25 CCCs, with hazard ratio and 95% confidence intervals shown for each factor. The favorable prognosis of cluster CCC-2 was independent of age and clinical stage.

To analyze expression profiles among the three histological subtypes in ovarian carcinomas, we performed microarray gene expression profiling in 55 ovarian carcinomas (25 CCCs, 14 ECs, 16 SCs) using HG-U133 Plus 2.0 arrays (Affymetrix). All RNA samples were obtained from tissue sections with a high proportion of carcinoma (>50%). The signal intensity was above the detection level with 13,830 probes out of the 54,675 probe set, and we further eliminated those transcripts expressed at a very low level, or those hybridized to a non-functional probe. Finally, 2640 transcripts were selected. Hierarchical clustering of the gene expression data showed a high degree of molecular structure defining three subtypes (Fig 2A). Cluster A was enriched with CCCs (19/21, 90%), cluster B with ECs (7/12, 58%), and cluster C with SCs (12/22, 55%) (Table 3). Thus, each cluster exhibited histology-dependent signatures in the expression array. Cluster A (CCC-enriched) showed specific clinicopathological characteristics (Table 3). The ratio of stage I/II tumors was significantly higher in cluster A (90%) than in clusters B and C (47%) (*P* = 0.0013).

**Table 3:** Clustering by expression arrays and clinicopathological characteristics.

	Cluster A(%)	Cluster B(%)	Cluster C(%)	p-value between Cluster A and Others
Histology				
CCC	19(90)	2(17)	4(18)	
EC	1(5)	7(58)	6(27)	p < 0.0001
SC	1(5)	3(25)	12(55)	
Stage				
I/II	19(90)	8(67)	8(36)	p = 0.0013
III/IV	2(10)	4(33)	14(64)	
Chemosensitivity				
Sensitive*	1(17)	2(100)	8(67)	p = 0.049
Resistant**	5(83)	0(0)	4(33)	
Emdometriosis				
Present	16(76)	4(33)	3(14)	p < 0.0001
Absent	5(24)	8(67)	19(86)	

Sensitive*: Complete response or Partial response.

Resistant**: Stable disease or Progressive disease.

p-value (by Fisher's exact test).

In 20 patients with measurable disease, the overall response rate (complete response + partial response by RECIST criteria) to platinum-taxane chemotherapy was 17% in cluster A, which was significantly lower than in clusters B and C (71%; *P* = 0.049). Endometriosis was more commonly observed in cluster A (76%), than in clusters B and C (21%; *P* < 0.0001) (Table 3). In accordance with previous reports [4,12–14], HIF-1 pathway genes and HNF-1beta were upregulated in cluster A, whereas p53 pathway genes were frequently deregulated in cluster C tumors (data not shown).

### Sub-clustering of CCC defines a poor prognostic clear cell signature that is associated with CIN-high status

As a proportion of the CCCs (23%) was classified as CIN-high, we further analyzed the CCC samples by gene expression profiling with HG-U133 Plus 2.0 microarrays. The signal intensity was above the detection level with 11,509 probes, and hierarchical clustering of gene expression data in 25 CCCs defined three subtypes and classified the tumors into three subgroups (clusters CCC-1, CCC-2, and CCC-3) (Fig 2B). Nine tumors (36%) were classified into CCC-1, ten (40%) into CCC-2, and six (24%) into CCC-3. We then addressed whether sub-clustering of CCC might be associated with clinicopathological findings. Four of 8 (50%) CCC-1 tumors were CIN-high, whereas only 1/12 was CIN-high in non-CCC-1 tumors. Progression free survival (PFS) was not significantly distinct between the CIN-high and CIN-low groups (Fig 2C). However, PFS for cluster CCC-2 was significantly better than that for clusters CCC-1 and CCC-3 (*P* = 0.042 by log-rank test, Fig 2D). The three-year PFS was 100% in CCC-2, but only 60% in CCC-1 and CCC-3 (Fig 2D and 2E).

To determine whether sub-classification by hierarchical clustering is reproducible by another method, we performed k-means clustering in these 25 CCC samples. k-means clustering revealed that 19 samples classified as CCC-1/CCC-2 by hierarchical clustering were clearly separated from 6 CCC-3 samples (S3A Fig). In addition, the k-means clustering for 3 k groups was identical to the hierarchical clustering of CCC-1, CCC-2, and CCC-3 (S3B Fig).

*PIK3CA* alterations (mutations and/or CNAs) were detected in 14 of 22 CCCs (64%), and loss of ARID1A expression was detected in 11 of 21 (52%) CCCs. Coexistent alterations of *PIK3CA* and *ARID1A* were detected in clusters CCC-1 and CCC-3 (7 of 11, 67%), but not in CCC-2 (0/10; *P* = 0.0039) (Table 4). CCC-1 and CCC-3 showed similar PFS (Fig 2E). However, early staged (stage I/II) tumors were more frequent in CCC-1 (9 of 9, 100%), compared with CCC-3 (3 of 6, 50%) (*P* = 0.044 by Fisher’s exact test) (Table 4).

**Table 4:** Correlation between clustering by expression arrays, genetic alterations and clinicopathological characteristics in CCC.

	CCC-1(%)	CCC-2(%)	CCC-3(%)	CCC-1/3(%)	CCC-2(%)	p-value
Stage						
I/II	9 (100)	8 (80)	3 (50)	12 (80)	8(80)	
III/IV	0 (0)	2 (20)	3 (50)	3 (20)	2(20)	1
Chemosensitivity						
Sensitive*	0 (0)	1 (100)	1 (33)	1 (17)	1 (100)	
Resistant**	3 (100)	0 (0)	2 (67)	5 (83)	0 (0)	0.29
CIN						
High	4 (50)	1 (11)	0 (0)	4 (36)	1 (11)	
Low/Negative	4 (50)	8 (89)	3 (100)	7 (64)	8 (89)	0.32
Mutation and/or CNA in PIK3CA						
Present	8 (89)	4 (40)	2 (50)	10 (77)	4 (40)	
Absent	1 (11)	6 (60)	2 (50)	3 (23)	6 (60)	0.1
ARID1A decreased expression						
Present	7 (78)	2 (20)	1 (50)	8 (73)	2 (20)	
Absent	2 (22)	8 (80)	1 (50)	3 (27)	8 (80)	0.03
Coexistent alterations in PIK3CA and ARID1A						
Present	6 (67)	0 (0)	1 (50)	7 (67)	0 (0)	
Absent	3 (33)	10 (100)	1 (50)	4 (33)	10 (100)	0.0039
Recurrence						
Present	3 (33)	0 (0)	2 (33)	3 (33)	0 (0)	
Absent	6 (67)	10 (100)	4 (67)	6 (67)	10 (100)	0.087

Sensitive*: Complete response or Partial response.

Resistant**: Stable disease or Progressive disease.

All three CCC-1 patients with recurrence (after >6 month treatment-free intervals) were resistant to platinum-based chemotherapy for recurrent tumors, suggesting an association with chemoresistance. Although the sample size is small, univariate analysis using stage, age, and gene expression clustering revealed that cluster CCC-2 alone was associated with favorable prognosis (Fig 2F). In addition, multivariate analysis revealed that cluster CCC-2 was an independent favorable prognostic factor in this setting (*P* = 0.010) (Fig 2F).

**Fig 3:**
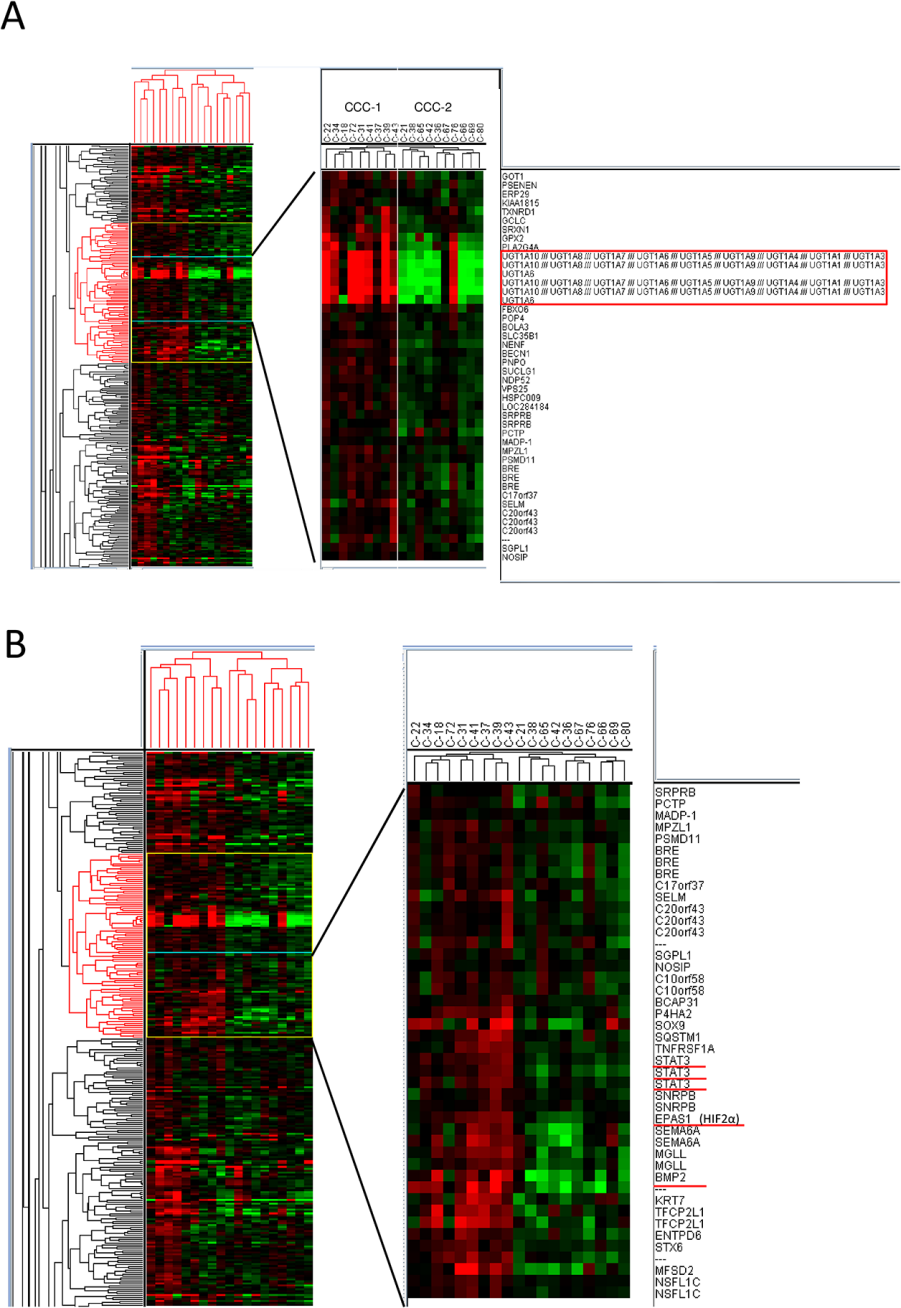
Integrated analyses reveal a poor-prognostic clear cell signature associated with high chromosomal instability. Comparison of clear cell carcinoma (CCC) clusters CCC-1 and CCC-2 by gene expression profiling. (A) *UGT1A6* and *UGT1A10* were significantly upregulated in CCC-1 compared with CCC-2. (B) The cluster of genes upregulated in CCC-1 compared with CCC-2. The cluster includes *STAT3* and *HIF2A*.

Lastly, we focused on pathway genes to further clarify the characteristics in each cluster. Comparison between the CCC-1 and CCC-2 clusters showed that *UGT1A* genes were highly upregulated in CCC-1 compared with CCC-2 (Fig 3A). In addition, *STAT3* and *EPAS1/HIF2A* (HIF-2α) were highly upregulated in CCC-1 compared with CCC-2 (Fig 3B). The expression levels of *UGT1A* in CCC-1 and CCC-2 were confirmed by real-time RT-PCR, using specific primers for either *UGT1A6* or *UGT1A10*. Both *UGT1A6* and *UGT1A10* were downregulated in all six CCC-2 samples tested, whereas 3 of 5 CCC-1 samples showed upregulation of these genes (S4 Fig). A comparison of pathway genes differentially expressed between the CCC-2 and CCC-3 clusters suggested that several oncogenes, including *PSAT1*, *CCNE1*, and *PAX8*, tended to be upregulated in the CCC-2 samples, compared with CCC-3 samples (S5A Fig). In contrast, extracellular matrix genes, including *COL5A2*, *COL10A1*, *COL11A1*, and *MMP2*, tended to be downregulated in the CCC-2 samples, compared with CCC-3 samples (S5B Fig).

## Discussion

In this study, the characteristics of CNAs and expression profiles were examined in ovarian cancers with a particular focus on (i) the differences between CCC and SC or EC, (ii) ‘hot spot’ CNA loci in each histological type, and (iii) sub-clustering of CCC and its association with prognosis.

Our analyses by SNP arrays showed that CIN status is significantly distinct among the histological subtypes. In agreement with a previous report [18, 27], significantly fewer CNAs were observed in CCCs than in SCs. We also observed that 8q amplification was common in both serous and clear cell histotypes. In this study, we show for the first time that the types, as well as the number, of CNAs were greatly distinct between CCC and SC. The ratio of whole-arm CNAs was significantly higher in CCCs, especially in chromosomes from 1p to 16q. As whole-arm CNAs are associated with mitotic instability [37], this molecular characteristic may represent a part of the tumor biology of CCC, and each CNA might be less associated with the aberrant expression of cancer related genes. On the other hand, focal CNAs at the loci of cancer related genes were significantly more frequent in SC than in CCC.

We focused on the loci of *BRCA* genes in SC, as the locus of *BRCA1* (17q21.2) is in the vicinity of that of *NF1* (17q11.2), while *BRCA2* (13q13.2) is located in the same chromosome as *RB1* (13q14.2). Our data revealed that LOH of *BRCA1/2* genes generally occurs concurrently with the LOH of *TP53*, *NF1*, and/or *RB1* in SCs. Although genetic mutations and hypermethylation of *BRCA*1 and *BRCA2* have been reported to be mutually exclusive [3], 62% of SCs possessed overlapping LOH of *BRCA1* and *BRCA2* in this study. Our data imply that haploinsufficiency of both *BRCA* genes might cooperatively impair the homologous recombination pathway, and that “BRCAness” might be a more frequent event in sporadic SC, as well as BRCA-mutated SC. There have been several clinical trials reporting the efficacy of poly-ADP ribose polymerase (PARP) inhibitors in ovarian serous adenocarcinomas [38,39]. SCs with overlapping LOH of *BRCA1* and *BRCA2* might be good candidates for PARP inhibitors. In the TCGA analyses, the mutation ratio of *BRCA1* and/or *BRCA2* was 20%, while those of *NF1* and *RB1* were only 4% and 2%, respectively [3]. In addition, the deletion of the loci of *NF1* and *RB1* was reported as only 8% and 8%, respectively [3]. Our data suggest that the focal CNAs affect the key tumor suppressor genes in the Rb and the Ras signaling axes, particularly in SCs with BRCA alterations. Copy number gains, including the loci of the RTK–PI3K pathway genes, were also predominant in SC (S3 Table). Although CNAs have been well analyzed in high-grade SC [40,41], further study is warranted to clarify the association between BRCAness and the Rb and Ras signaling pathways.

Hierarchical clustering of 55 ovarian carcinomas by microarray analysis demonstrated histology-dependent expression signatures. These data are in agreement with previous findings [13–15]. Clusters A, B, and C predominantly included CCCs, ECs, and SCs, respectively. Although the ratio of stage I/II tumors was significantly higher in cluster A, this cluster showed poorer chemosensitivity than the others, suggesting the chemoresistant characteristics of CCC. In agreement with previous findings showing similarity between high-grade EC and high-grade SC [42], both ECs with a grade of 3 (poorly differentiated type) out of the 14 ECs studied were classified into CIN-high group and expression array cluster C (SC-enriched cluster). A recent study suggested that expression profiles and chromosomal instability might be predictive of prognosis in CCC and SC [20, 43]. Hence, we hypothesized that expression profiling might be useful to elucidate CCC subgroups with distinct prognoses. Significantly, one of the three CCC clusters (CCC-2) was associated with favorable prognosis. Although all nine tumors were stage I/II in CCC-1, the prognosis was worse than that of CCC-2. Taken together with the high ratio of CIN-high in CCC-1, CIN-high might be associated with chemoresistance and poor prognosis in CCC. Multivariate analysis revealed that the CCC-2 signature was an independent favorable prognostic factor in CCC. Thus, these sub-classifications might be useful for prediction of prognosis and chemosensitivity in CCC. Furthermore, the oncogenic mutation of *PIK3CA* is reported to activate STAT3 and IL-6 in an NF-κB-dependent manner [44]. Indeed, the CCC-1 cluster displaying a high ratio of *PIK3CA* mutation (8/9; 89%) is accompanied by an upregulation of STAT3. By comparing expression profiles between CCC-2 and CCC-3, E2F-RB pathway genes, such as *PSAT1*, *Pax8* and cyclin E1 (*CCNE1*) [45, 46] were upregulated in CCC-2. However, extracellular matrix and TGF-beta related genes, including *COL5A2*, *COL10A1*, *COL11A1*, and *MMP2* [47, 48], tended to be downregulated in CCC-2, compared with CCC-3. These data might be associated with the high ratio of advanced stage in CCC-3 (3 of 6 cases were stage III, IV). Further study is warranted to validate the expression profiles in each cluster. In addition, we found that the sub-classification in CCC was associated with *PIK3CA* and *ARID1A* alterations, which have not been reported in previous studies [27, 49]. Further analysis is warranted to establish the chemoresistant and poor prognostic subgroups in CCC.

In conclusion, we showed that CCCs are statistically more likely to exhibit whole-arm CNAs, and that focal LOH in SC correlates *BRCA1/2* genes with other major tumor suppressor genes, including *TP53*, *RB1*, and *NF1*. Our observations also suggest that some differentially expressed genes in CCC-2 (such as *UGT1A*, *STAT3*, *HIF2A*, E2F-RB and extracellular matrix genes) may serve as possibly favorable prognostic indicators. Most importantly, we established that CCC could be subclassified according to the gene expression profiles, which might be associated with prognosis and chemoresistance. Our study has several limitations, including a small sample size and a defined focus on *PIK3CA* gene mutations. As the number of CCC specimens (n = 25) was relatively small for hierarchical clustering by gene expression profiling, careful consideration is necessary to interpret the prognostic impact of the subgroups in CCC. In addition, transcriptomic subclassification can be influenced by tumor purity itself. As publically available expression array data are limited in CCC, further validation in a large cohort is warranted to determine prognostically significant subgroups and identify representative gene sets for CCC prognosis. A more comprehensive analysis, including whole-exome sequencing and chemosensitivity profiling, is warranted. Nevertheless, we believe these results are significant and provide a substantial foundation for the continued exploration of CCC profiling.

## Supporting Information

S1 Fig The representative SNP array ‘karyograms’ of each tumor. We divided the samples according to the number of CNAs into three subgroups: CIN-high (with nine or more CNAs), CIN-low (with one to eight CNAs) and CIN-negative (without any CNAs).
(PPTX)

S2 Fig Overview of genomic imbalance sorted by the histological subtypes. (Right) Frequency of gains and losses is compared among the 3 histological subtypes. Gains, losses and copy number neutral LOH (CNN-LOH) were shown in separate colors.
(PPTX)

S3 Fig k-means clustering in 25 CCCs. k-means clustering was performed as follows: (i) changing the sample order 1,000 times by selecting randomly 3,000 genes, (ii) identifying samples that were classified in the same cluster together, and (iii) repeating steps (i) and (ii) for 2 to 10 k groups. The error values according to non-negative matrix factorization for 2 k and 3 k were 0.191 and 1.044, respectively, which were lower than any other (4 k to 10 k) group. (A) 2 k clustering in 25 CCCs. The left cluster consists of CCC1 and CCC-2 (n = 19) and the right cluster consists of CCC-3 (n = 6). (B) 3 k clustering in 25 CCCs. The left, middle, and right clusters correspond to CCC-1, CCC-2, and CCC-3, respectively.
(PPTX)

S4 Fig Real-time RT-PCR of *UGT1A*. Real-time RT-PCR validation of differentially expressed genes (*UGT1A6* and *UGT1A10*) identified in CCC-1 and CCC-2 clusters in the expression array. Quantitative real-time PCR was performed with 5 CCC-1 tumor samples and 6 CCC-2 tumor samples, using total RNAs and primers specific for *UGT1A6* (upper lanes) and *UGT1A10* (lower lanes) that were downregulated in CCC-2 (compared with CCC-1), based on expression array results. Relative gene expression levels in quantitative PCR were first normalized to the reference gene (*GAPDH*), and then calculated as fold-changes relative to the sample in which the gene expression level was the lowest.
(PPTX)

S5 Fig Comparison of clear cell carcinoma (CCC) clusters CCC-2 and CCC-3 by gene expression profiling. (A) The cluster of genes upregulated in CCC-2, compared with CCC-3. The cluster includes *PSAT1*, *PAX8*, and *CCNE1*. (B) The cluster of genes downregulated in CCC-2, compared with CCC-3. The cluster includes *COL5A2*, *COL10A1*, *COL11A1*, and *MMP2*.
(PPTX)

S1 Table Patient characteristics of 80 ovarian carcinomas. (XLSX)

S2 Table Chromosomal copy number alterations in each chromosome. (PPTX)

S3 Table The regions of copy number alterations and cancer-related genes. (PPTX)
