# Correction: Integrated Copy Number and Expression Analysis Identifies Profiles of Whole-Arm Chromosomal Alterations and Subgroups with Favorable Outcome in Ovarian Clear Cell Carcinomas

**DOI:** 10.1371/journal.pone.0132751

**Published:** 2015-07-06

**Authors:** Yuriko Uehara, Katsutoshi Oda, Yuji Ikeda, Takahiro Koso, Shingo Tsuji, Shogo Yamamoto, Kayo Asada, Kenbun Sone, Reiko Kurikawa, Chinami Makii, Otoe Hagiwara, Michihiro Tanikawa, Daichi Maeda, Kosei Hasegawa, Shunsuke Nakagawa, Osamu Wada-Hiraike, Kei Kawana, Masashi Fukayama, Keiichi Fujiwara, Tetsu Yano, Yutaka Osuga, Tomoyuki Fujii, Hiroyuki Aburatani

There are errors in the Data Availability statement. The correct statement is: The expression array data are freely available in the GEO website (The accession number is GSE65986 in GEO website (http://www.ncbi.nlm.nih.gov/geo/query/acc.cgi?acc=GSE65986). The SNP array data are available from NBDC (National Bioscience Database Center) website under controlled access (http://biosciencedbc.jp/en/). The NBDC number is hum0030, and the JGA (Japanese Genotype-phenotype Archive) accession number is JGA00000000022.
