# Supplementary material for: Integrated Copy Number and Expression Analysis Identifies Profiles of Whole-Arm Chromosomal Alterations and Subgroups with Favorable Outcome in Ovarian Clear Cell Carcinomas
Source: PLoS One. 2015 Jun 4;10(6):e0128066. doi: 10.1371/journal.pone.0128066 (PMC4456367; doi:10.1371/journal.pone.0128066)
Supplement: S1 Fig — We divided the samples according to the number of CNAs into three subgroups: CIN-high (with nine or more CNAs), CIN-low (with one to eight CNAs) and CIN-negative (without any CNAs). (PPTX) [file pone.0128066.s001.pptx]

## Slide 1
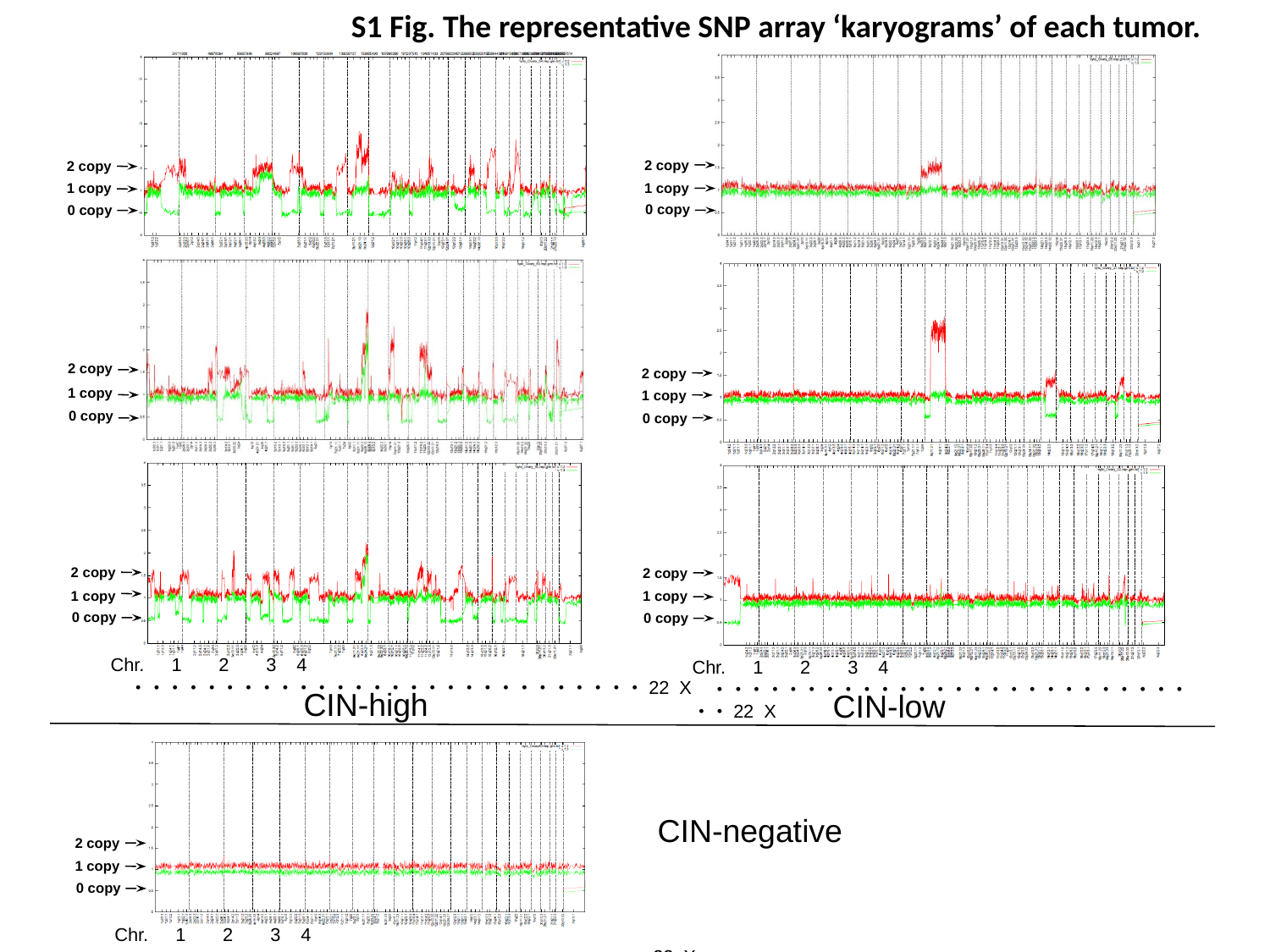

S1 Fig. The representative SNP array ‘karyograms’ of each tumor.
2 copy
1 copy
0 copy
2 copy
1 copy
0 copy
2 copy
1 copy
0 copy
Chr.　1 　 2 　3 4 　・・・・・・・・・・・・・・・・・・・・・・・・・・・・22 X
2 copy
1 copy
0 copy
2 copy
1 copy
0 copy
2 copy
1 copy
0 copy
Chr.　1 　 2 　3 4 　・・・・・・・・・・・・・・・・・・・・・・・・・・・・22 X
CIN-low
CIN-high
Chr.　1 　 2 　3 4 　・・・・・・・・・・・・・・・・・・・・・・・・・・・・22 X
2 copy
1 copy
0 copy
CIN-negative
