# Supplementary material for: Integrated Copy Number and Expression Analysis Identifies Profiles of Whole-Arm Chromosomal Alterations and Subgroups with Favorable Outcome in Ovarian Clear Cell Carcinomas
Source: PLoS One. 2015 Jun 4;10(6):e0128066. doi: 10.1371/journal.pone.0128066 (PMC4456367; doi:10.1371/journal.pone.0128066)
Supplement: S2 Fig — (Right) Frequency of gains and losses is compared among the 3 histological subtypes. Gains, losses and copy number neutral LOH (CNN-LOH) were shown in separate colors. (PPTX) [file pone.0128066.s002.pptx]

## Slide 1
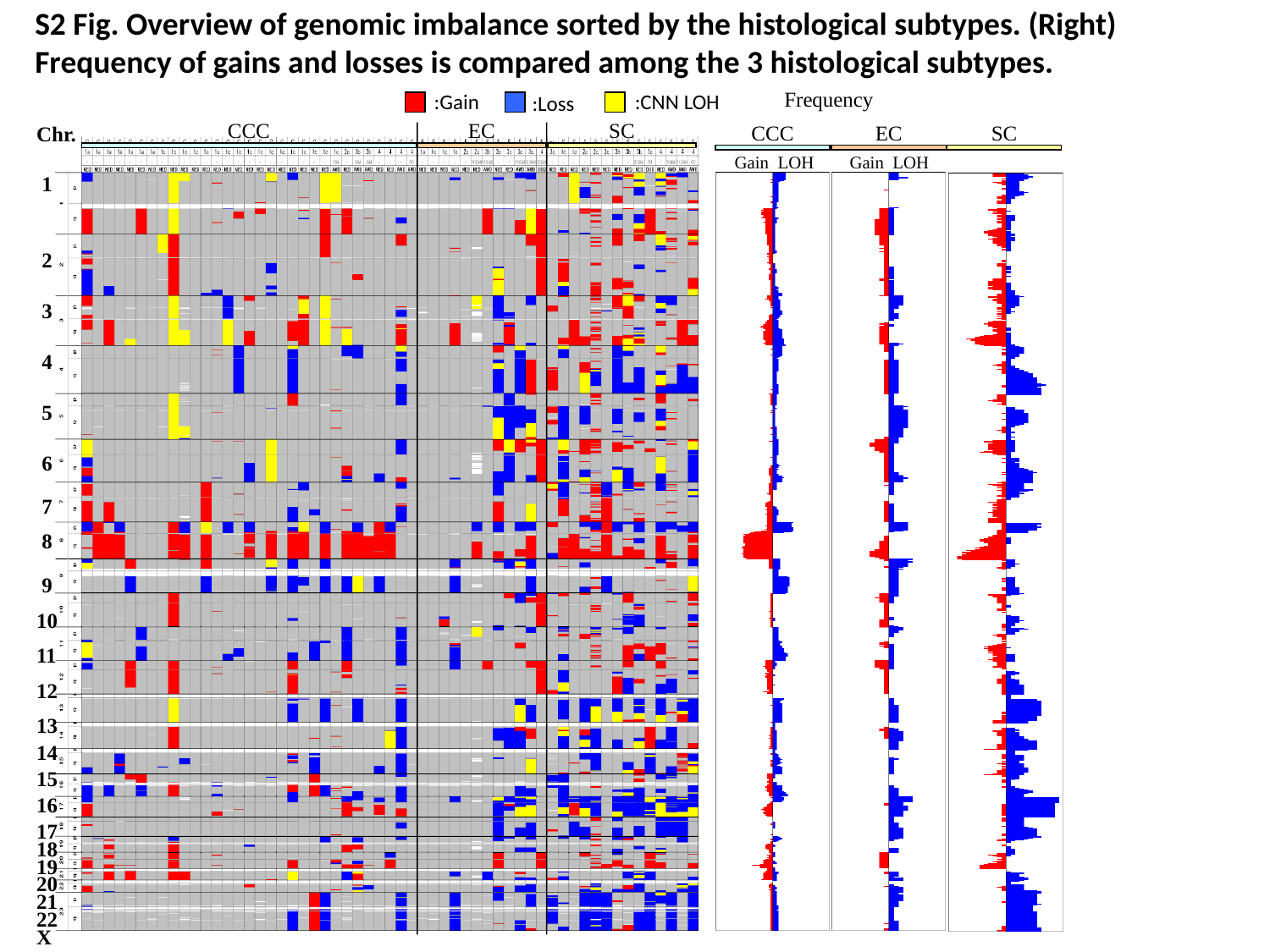

S2 Fig. Overview of genomic imbalance sorted by the histological subtypes. (Right) Frequency of gains and losses is compared among the 3 histological subtypes.
Fisher’s exact test
p-value
Gain
LOH
Frequency
CCC
EC
Gain
LOH
Gain
LOH
EC
SC
CCC
SC
Chr.
 1
 2
 3
 4
 5
 6
 7
 8
 9
10
11
12
13
14
15
16
17
18
19
20
21
22
X
:Gain
:CNN LOH
:Loss
