# Supplementary figures and images for: Integrated Copy Number and Expression Analysis Identifies Profiles of Whole-Arm Chromosomal Alterations and Subgroups with Favorable Outcome in Ovarian Clear Cell Carcinomas
Source: PLoS One. 2015 Jun 4;10(6):e0128066. doi: 10.1371/journal.pone.0128066 (PMC4456367; doi:10.1371/journal.pone.0128066)

## Slide 1
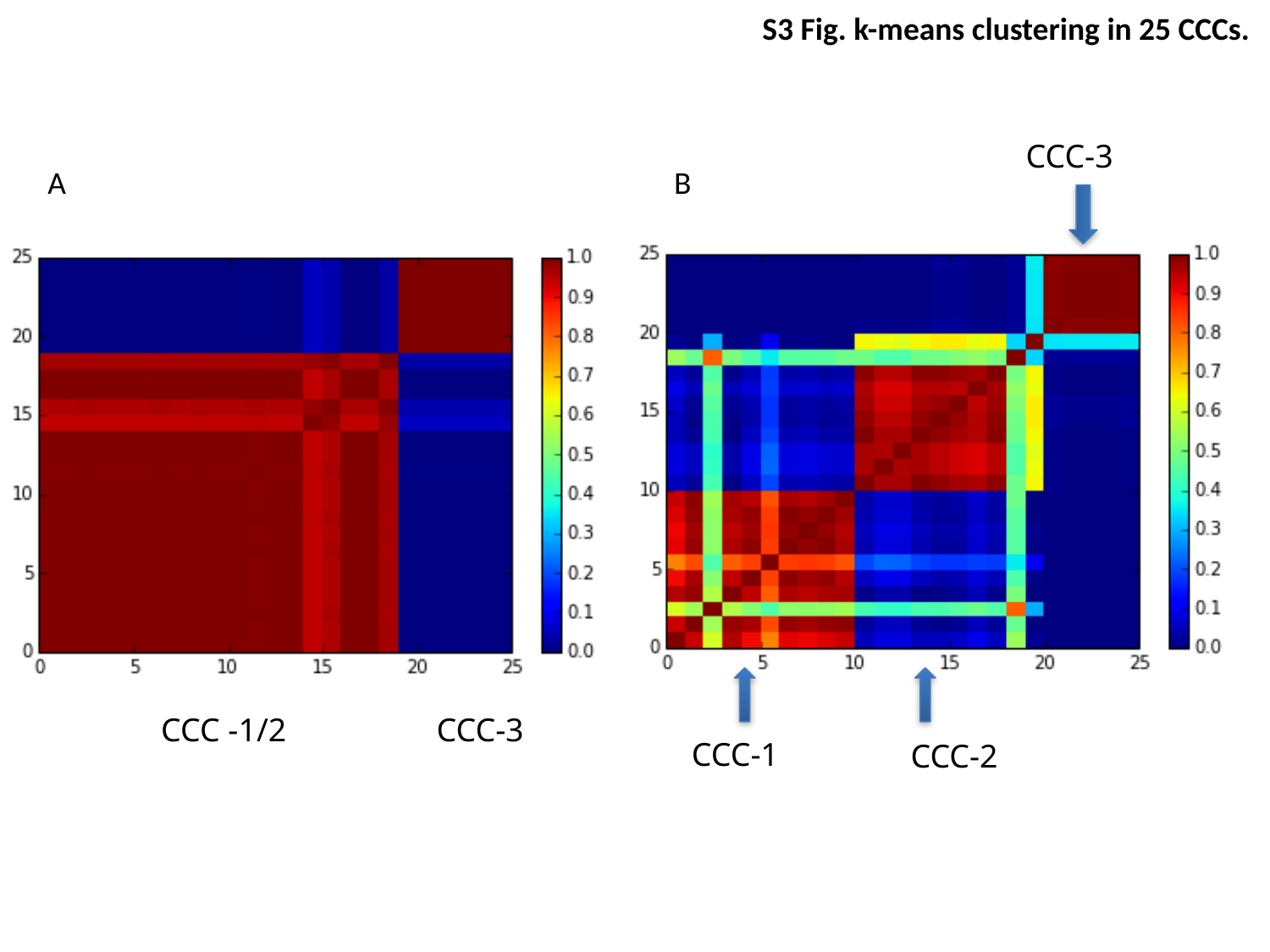

S3 Fig. k-means clustering in 25 CCCs.
CCC-3
A
B
CCC -1/2
CCC-3
CCC-1
CCC-2

Supplement: S3 Fig — k-means clustering was performed as follows: (i) changing the sample order 1,000 times by selecting randomly 3,000 genes, (ii) identifying samples that were classified in the same cluster together, and (iii) repeating steps (i) and (ii) for 2 to 10 k groups. The error values according to non-negative matrix factorization for 2 k and 3 k were 0.191 and 1.044, respectively, which were lower than any other (4 k to 10 k) group. (A) 2 k clustering in 25 CCCs. The left cluster consists of CCC1 and CCC-2 (n = 19) and the right cluster consists of CCC-3 (n = 6). (B) 3 k clustering in 25 CCCs. The left, middle, and right clusters correspond to CCC-1, CCC-2, and CCC-3, respectively. (PPTX) [file pone.0128066.s003.pptx]

## Slide 1
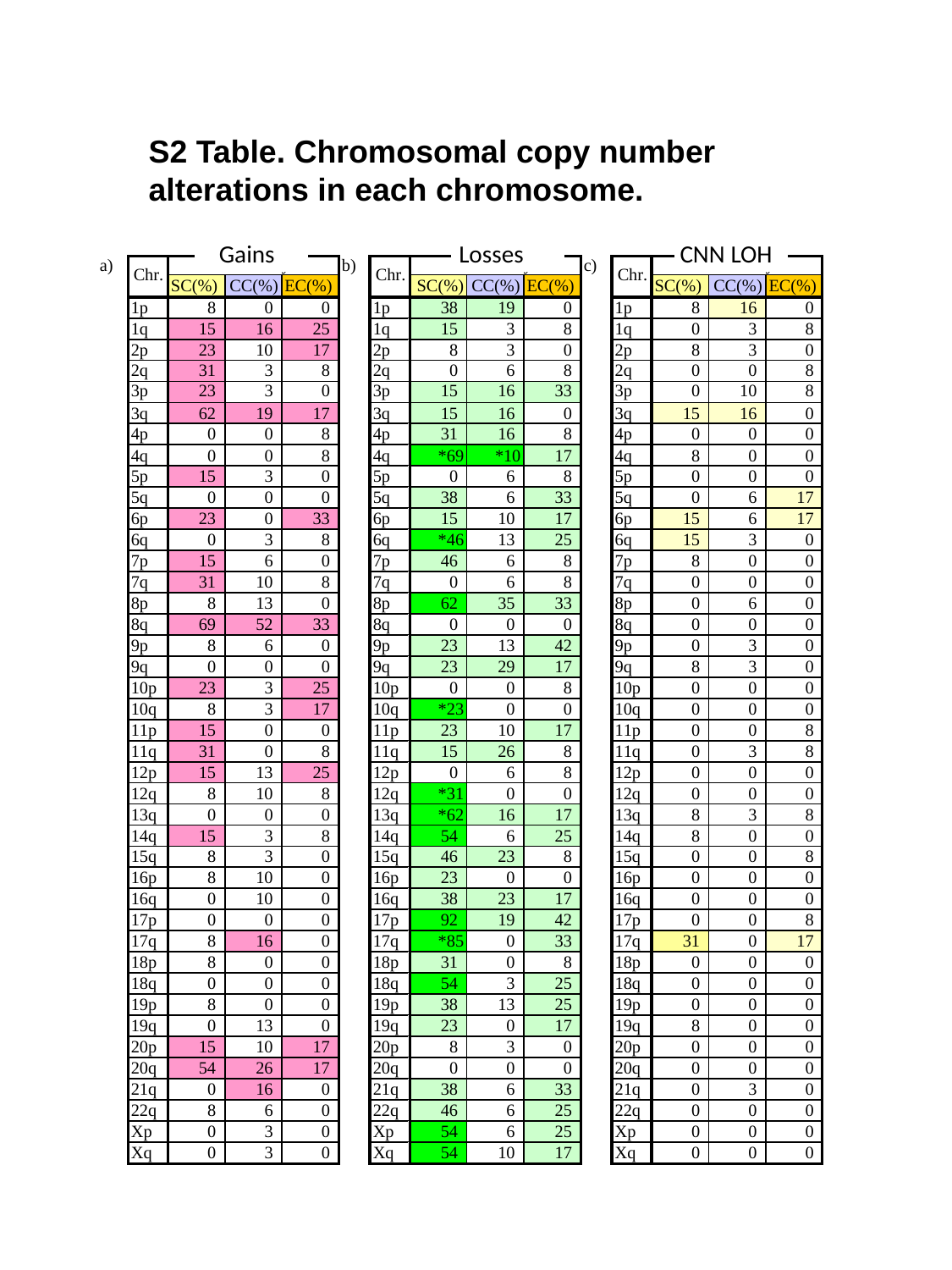

S2 Table. Chromosomal copy number alterations in each chromosome.
Gains
Losses
CNN LOH

Supplement: S2 Table — (PPTX) [file pone.0128066.s007.pptx]
