# Supplementary material for: Integrated Copy Number and Expression Analysis Identifies Profiles of Whole-Arm Chromosomal Alterations and Subgroups with Favorable Outcome in Ovarian Clear Cell Carcinomas
Source: PLoS One. 2015 Jun 4;10(6):e0128066. doi: 10.1371/journal.pone.0128066 (PMC4456367; doi:10.1371/journal.pone.0128066)
Supplement: S4 Fig — Real-time RT-PCR validation of differentially expressed genes (UGT1A6 and UGT1A10) identified in CCC-1 and CCC-2 clusters in the expression array. Quantitative real-time PCR was performed with 5 CCC-1 tumor samples and 6 CCC-2 tumor samples, using total RNAs and primers specific for UGT1A6 (upper lanes) and UGT1A10 (lower lanes) that were downregulated in CCC-2 (compared with CCC-1), based on expression array results. Relative gene expression levels in quantitative PCR were first normalized to the reference gene (GAPDH), and then calculated as fold-changes relative to the sample in which the gene expression level was the lowest. (PPTX) [file pone.0128066.s004.pptx]

## Slide 1
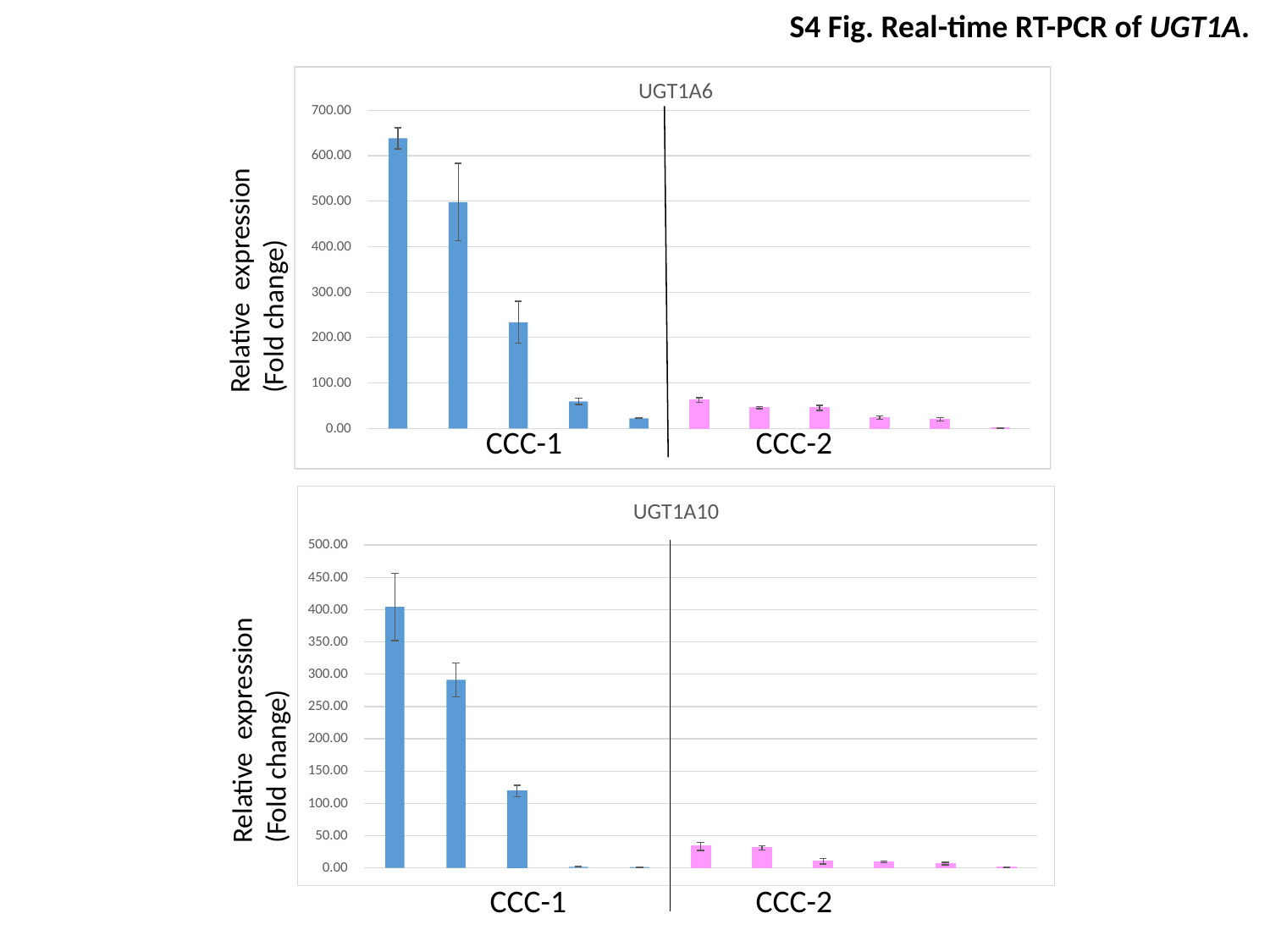

S4 Fig. Real-time RT-PCR of UGT1A.
Relative expression (Fold change)
CCC-1
CCC-2
Relative expression (Fold change)
CCC-1
CCC-2
