# Supplementary material for: Integrated Copy Number and Expression Analysis Identifies Profiles of Whole-Arm Chromosomal Alterations and Subgroups with Favorable Outcome in Ovarian Clear Cell Carcinomas
Source: PLoS One. 2015 Jun 4;10(6):e0128066. doi: 10.1371/journal.pone.0128066 (PMC4456367; doi:10.1371/journal.pone.0128066)
Supplement: S5 Fig — (A) The cluster of genes upregulated in CCC-2, compared with CCC-3. The cluster includes PSAT1, PAX8, and CCNE1. (B) The cluster of genes downregulated in CCC-2, compared with CCC-3. The cluster includes COL5A2, COL10A1, COL11A1, and MMP2. (PPTX) [file pone.0128066.s005.pptx]

## Slide 1
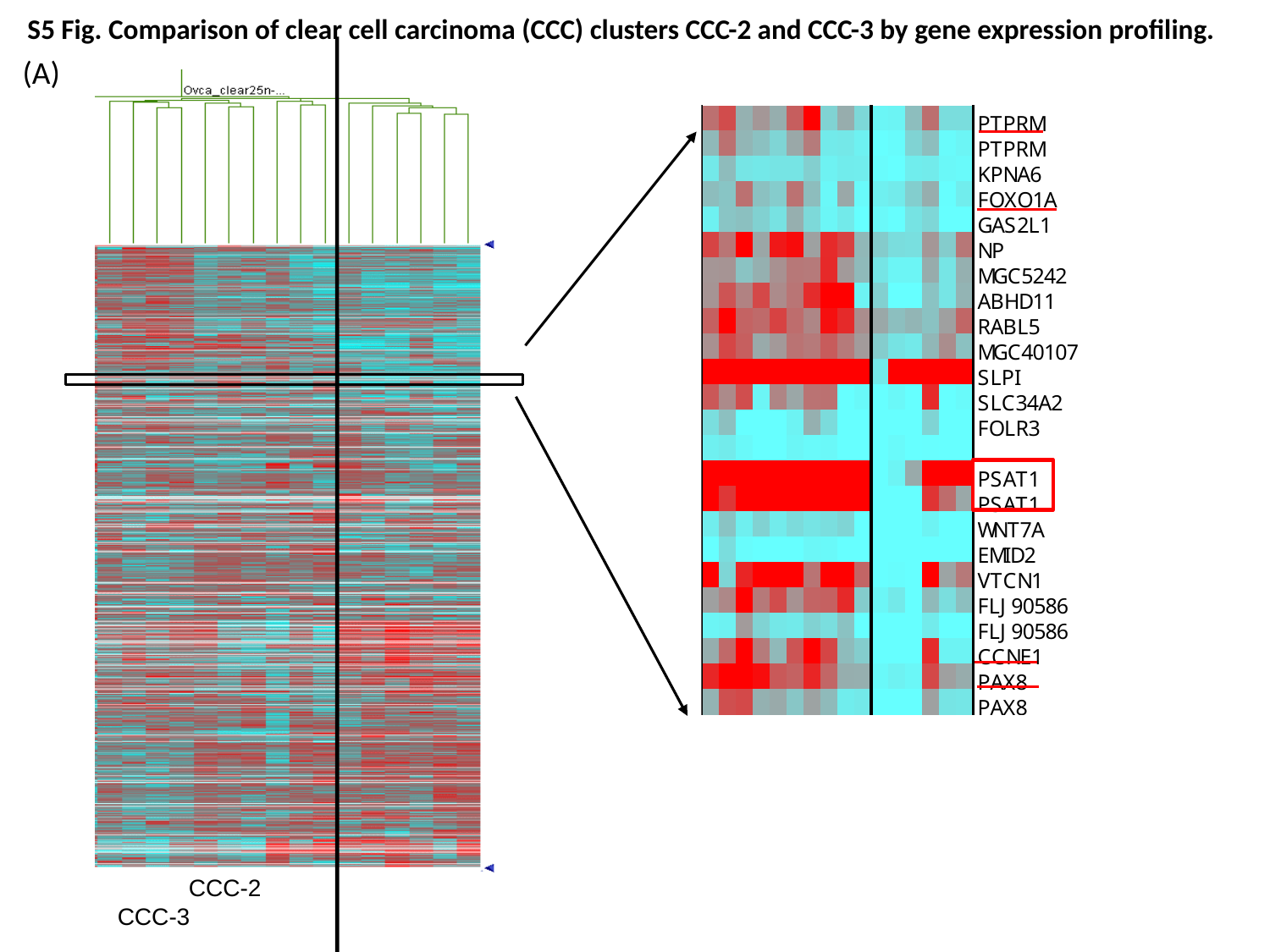

S5 Fig. Comparison of clear cell carcinoma (CCC) clusters CCC-2 and CCC-3 by gene expression profiling.
(A)
A
　　　　CCC-2　　　　　　　　　　CCC-3

## Slide 2
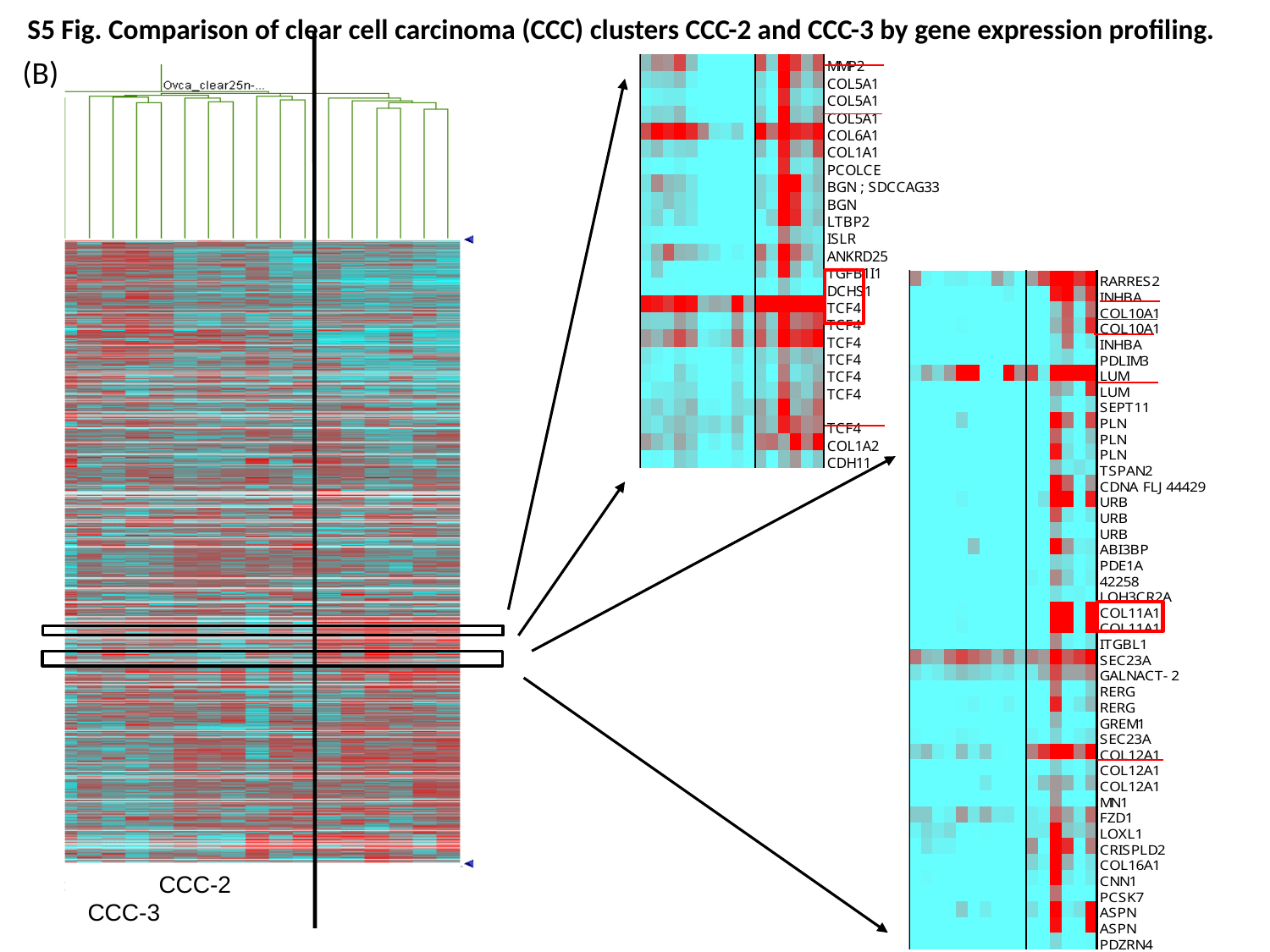

S5 Fig. Comparison of clear cell carcinoma (CCC) clusters CCC-2 and CCC-3 by gene expression profiling.
(B)
A
　　　　CCC-2　　　　　　　　　　CCC-3
