# Supplementary material for: Integrated Copy Number and Expression Analysis Identifies Profiles of Whole-Arm Chromosomal Alterations and Subgroups with Favorable Outcome in Ovarian Clear Cell Carcinomas
Source: PLoS One. 2015 Jun 4;10(6):e0128066. doi: 10.1371/journal.pone.0128066 (PMC4456367; doi:10.1371/journal.pone.0128066)
Supplement: S3 Table — (PPTX) [file pone.0128066.s008.pptx]

## Slide 1
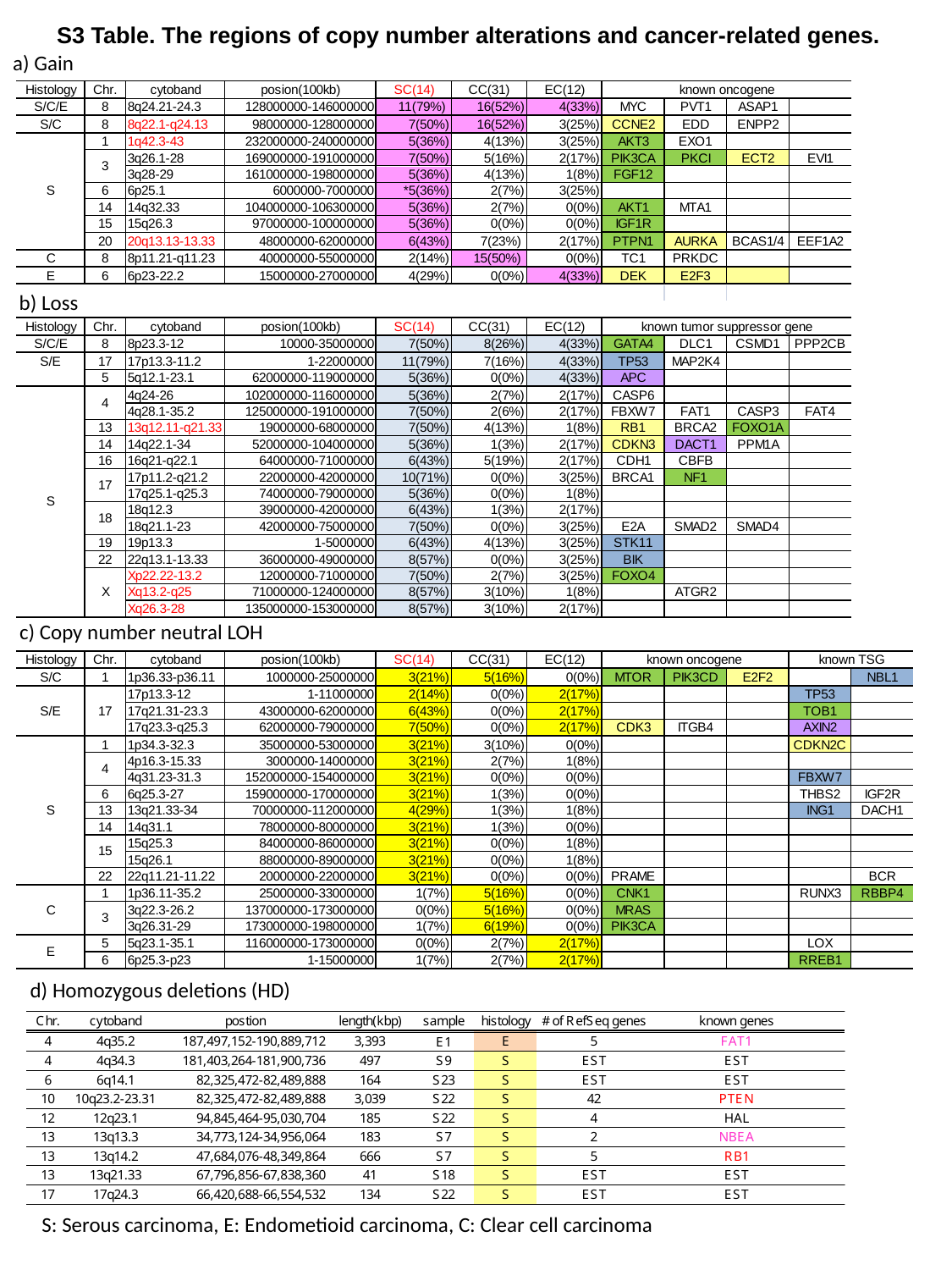

S3 Table. The regions of copy number alterations and cancer-related genes.
a) Gain
b) Loss
c) Copy number neutral LOH
d) Homozygous deletions (HD)
S: Serous carcinoma, E: Endometioid carcinoma, C: Clear cell carcinoma
